# Multi-targeted inhibition of NF-κB signaling underlies the anti-osteoarthritic effects of BGJXF and its key component dehydrocorydaline

**DOI:** 10.3389/fmed.2025.1676071

**Published:** 2026-01-09

**Authors:** Jingyu Shen, Zhenpeng Bin, Yunfu Shen, Fang Xie, Xincheng Zhang, Xuyi Tan, Hui Xu

**Affiliations:** 1 Hunan Provincial Hospital of Integrated Traditional Chinese and Western Medicine, Changsha, China; 2 Graduate School, Hunan University of Chinese Medicine, Changsha, China

**Keywords:** Bu Gan Jian Xi Fang, knee osteoarthritis, dehydrocorydaline, NF-κB pathway, network pharmacology

## Abstract

**Background:**

Knee osteoarthritis (KOA) poses a significant global health challenge due to its high prevalence and the limited availability of effective treatment options. This study aims to elucidate the multi-target therapeutic mechanisms of bu gan jian xi fang (BGJXF), a traditional Chinese medicine (TCM) formula, in the treatment of KOA.

**Methods:**

The study employed integrated liquid chromatography-mass spectrometry (LC–MS) analysis to identify the chemical constituents of BGJXF. Network pharmacology was subsequently utilized to predict potential therapeutic targets shared by BGJXF and KOA. Enrichment analysis was conducted to identify key pathways, while molecular docking was used to assess the binding affinities of principal components to core targets. These mechanisms were further validated through *in vitro* experiments, measuring NF-κB p65 and TNF-*α* levels in LPS-stimulated human chondrocytes treated with dehydrocorydaline. Additionally, *in vivo* validation was performed using a papain-induced KOA rat model to evaluate serum cytokines (IL-1β, IL-6), histopathological cartilage damage and inflammation, and the tissue microenvironment via immunofluorescence following BGJXF treatment.

**Results:**

LC–MS analysis identified 91 chemical constituents in BGJXF. Network pharmacology predicted 62 shared therapeutic targets, which were significantly enriched in the AGE-RAGE and HIF-1 signaling pathways. Molecular docking identified dehydrocorydaline as a key component with strong binding affinities to IL-6, BCL2, MMP9, and CCND1. *In vitro*, dehydrocorydaline effectively suppressed LPS-induced overexpression of NF-κB p65 and TNF-*α* in chondrocytes. *In vivo*, BGJXF treatment significantly reduced serum IL-1β and IL-6 levels, mitigated cartilage structural damage and inflammatory cell infiltration as shown by histopathological analysis, and modulated tissue heterogeneity in the KOA rat model.

**Conclusion:**

Collectively, these findings indicate that BGJXF, driven by dehydrocorydaline, exerts chondroprotective and anti-inflammatory effects primarily through inhibition of the NF-κB pathway via a multi-target mechanism. The overall therapeutic efficacy of BGJXF may be mediated by its modulation of the dual-pathology axis of “glycometabolic stress-hypoxic injury,” which is formed by the AGE-RAGE and HIF-1 signaling pathways. This study lays a pharmacological foundation for the application of BGJXF as a multi-target therapeutic strategy for the management of KOA.

## 1 Introduction

Knee osteoarthritis (KOA) is a chronic, whole-joint disorder characterized primarily by degenerative changes in the articular cartilage. It involves all joint tissues, including meniscal degeneration and inflammation of the infrapatellar fat pad, in addition to synovial inflammation, subchondral bone sclerosis, osteophyte formation, and progressive cartilage degradation (1, 2). With the acceleration of global population aging, the prevalence of KOA is showing a significant upward trend. In China, the current prevalence rate is 25.51% (3), and driven by socioeconomic development, population aging, and increased life expectancy, this prevalence is projected to rise annually (4). The disease not only causes persistent joint pain and morning stiffness but also leads to reduced knee range of motion, abnormal gait, and muscle atrophy. In severe cases, joint deformity and functional loss can occur, significantly restricting patients’ activities of daily living, diminishing quality of life, and imposing a substantial socioeconomic burden (4, 5). Consequently, in-depth research into the pathophysiological mechanisms and therapeutic strategies for KOA holds significant clinical importance.

Currently, clinical interventions for KOA mainly adopt a stepwise treatment approach, but there are significant limitations in terms of safety and efficacy. In terms of drug treatment, nonsteroidal anti-inflammatory drugs (NSAIDs) can relieve pain in the short term, but long-term use carries the risk of serious complications, including renal impairment, cardiovascular events, and gastrointestinal bleeding (6–8). Although intra-articular injections of corticosteroids can quickly suppress synovial inflammation, repeated injections may accelerate cartilage matrix degradation and increase the risk of joint infection (9). Hyaluronic acid (viscosupplementation) injections, which have been widely used in recent years, can improve joint function through lubrication; however, multiple evidence-based studies have shown that they have no significant effect on cartilage repair, and the duration of treatment is usually no more than 6 months (10, 11). In the field of physical therapy, although exercise therapy is recommended as a first-line intervention by the American college of rheumatology (ACR) (12), Patient compliance with exercise interventions is often low in people with KOA (13), and for those with severe structural joint damage, the therapeutic benefit of exercise may be further diminished (14). Other physical treatments, such as extracorporeal shock wave therapy (ESWT) and ultrasound, are hampered by large individual differences in efficacy and unclear mechanisms of action (15–17). When conservative treatment is ineffective, surgical intervention often becomes the only option for treating advanced KOA (18, 19). However, the 2021 osteoarthritis research society international (OARSI) guidelines re-evaluated the clinical value of arthroscopic debridement and stated that it is “not recommended for routine use” due to its lack of clear cartilage regeneration benefits (20). Although total joint replacement provides a clear treatment option for end-stage disease, its complication rate is relatively high, including prosthesis loosening and infection, while the cost of surgery and long rehabilitation period also impose a heavy burden on elderly patients (21). The common shortcomings of these treatments are the failure to effectively target the core pathological process of KOA - cartilage degeneration and metabolic imbalance, and the lack of multi-target synergistic intervention strategies. Thus, it is critical to find safer and more effective drugs to intervene in the progression of KOA from early to mid-stage.

Traditional Chinese medicine (TCM) is renowned worldwide for its unique multi-target and multi-pathway modes of action (22, 23). A large amount of clinical and research evidence has demonstrated the key role of TCM in the treatment of KOA. In addition, the simplicity, efficacy, accessibility, and low cost of TCM have inspired researchers to explore TCM interventions and give full play to its holistic conditioning advantages. BGJXF is an empirical clinical prescription for the treatment of early and mid-stage KOA developed by Professor Xiangzhong Qiu, a renowned TCM expert in Hunan Province and a national-level famous TCM practitioner. BGJXF is derived from the classic prescription Bugan Tang, with the main ingredients being *Ziziphus jujuba*, *Radix Rehmanniae*, *Radix Achyranthis Bidentatae*, and *Radix Paeoniae Alba*. Its therapeutic concepts of “nourishing the liver and strengthening the tendons” and “removing blood stasis and unblocking the meridians” are consistent with TCM theories on KOA management. Although BGJXF has shown good efficacy in the treatment of KOA, its mechanism of action is not fully understood and lacks comprehensive analysis (24). In clinical practice, BGJXF is typically administered orally in the form of a decoction.

The application of the BGJXF formula is primarily concentrated in Hunan Province, where Professor Xiangzhong Qiu has practiced traditional Chinese medicine for over 30 years. According to clinical records, since 2010, this formula has been prescribed an average of more than 60 times per month in our hospital and collaborating institutions, although a comprehensive database on its usage frequency has not yet been established.

Importantly, according to the classical TCM theory of “liver deficiency and collateral impediment,” the pathogenesis of KOA is believed to originate from chronic insufficiency of liver blood, leading to poor nourishment of the sinews and vessels. This state further results in collateral blockage due to blood stasis and phlegm accumulation, ultimately manifesting as joint pain, stiffness, and functional impairment. Therefore, the therapeutic principle in TCM focuses on nourishing the liver and replenishing blood to restore tendon function, as well as activating blood and unblocking the collaterals to relieve pain. The formulation of BGJXF was guided by these classical concepts, aiming to address both liver blood deficiency and collateral obstruction, which are regarded as key pathological mechanisms in early and mid-stage KOA.

Therefore, this study aimed to systematically elucidate the therapeutic mechanisms of BGJXF in the management of knee osteoarthritis. Specifically, we sought to identify the key bioactive components, predict and validate their molecular targets and pathways, and assess the *in vivo* efficacy of BGJXF in experimental models of KOA. This work is expected to provide a scientific basis for the broader clinical application of BGJXF.

## 2 Materials and methods

A multi-disciplinary strategy integrating chemical profiling, network pharmacology, molecular docking, and animal experiments was employed to investigate the mechanisms of BGJXF in KOA.

### 2.1 Materials

Instrumentation: Ultra-high-performance liquid chromatography (UHPLC) system (UltiMate 3,000, Thermo Fisher Scientific, USA); high-resolution mass spectrometer (HRMS) (5,600 QTOF, AB Sciex, USA); chromatographic column (ACQUITY UPLC HSS T3, 1.8 μm, 2.1mm × 100 mm, Waters, USA); CO₂ incubator (Thermo Forma 311, Thermo Fisher Scientific, USA); electrophoresis system (Tanon EPS300, Tanon Science & Technology, China); chemiluminescence imaging system; centrifuges (Eppendorf 5415R, Eppendorf, Germany; Lu Xiangyi TDZ4-WS, China).

Drugs: The BGJXF formula consisted of the following Chinese herbal formula granules (all sourced from the Affiliated Hospital of Hunan Academy of Chinese Medicine): *Radix Paeoniae Alba* (Bai Shao) 30 g; *Radix Angelicae Dahuricae* (Bai Zhi) 10 g; *Fructus Hordei Germinatus Praeparatus* (Chao Mai Ya, fried barley sprout) 10 g; *Semen Plantaginis* (Che Qian Zi) 10 g; *Radix Angelicae Sinensis* (Dang Gui) 10 g; *Radix et Rhizoma Glycyrrhizae* (Gan Cao) 6 g; *Ramulus Cinnamomi* (Gui Zhi) 12 g; *Bombyx Batryticatus* (Jiang Can) 10 g; *Fructus Chaenomelis* (Mu Gua) 15 g; *Radix Cyathulae* (Chuan Niu Xi) 15 g; *Ramulus Mori* (Sang Zhi) 15 g; *Radix Rehmanniae Praeparata* (Shu Di Huang) 25 g; *Radix Rehmanniae Recens* (Sheng Di Huang) 15 g; *Semen Ziziphi Spinosae* (Suan Zao Ren) 20 g; *Rhizoma Alismatis* (Ze Xie) 10 g; *Scolopendra* (Wu Gong) 3 g. All herbal granules were authenticated according to the Chinese Pharmacopoeia (2020 edition). The prescribed herbs were weighed and combined according to the clinical prescription, and decocted in distilled water at the Experimental Center of Hunan Academy of Traditional Chinese Medicine. Decoction was performed twice (each time with a tenfold volume of water, boiling for 30 min per session). The combined extracts were filtered and concentrated to obtain the BGJXF aqueous decoction for subsequent experiments.

Reagents: Acetonitrile (HPLC grade, Merck, Darmstadt, Germany); methanol (HPLC grade, Merck, Darmstadt, Germany); formic acid (HPLC grade, CNW Technologies, Shanghai, China); ultrapure water (Merck, Darmstadt, Germany); ammonium acetate (HPLC grade, CNW Technologies, Shanghai, China); lipopolysaccharide (LPS, from *E. coli* O111: B4, Solarbio, Beijing, China; Cat# L8880); dehydrocorydaline (Ambeed, Arlington Heights, IL, USA; Cat# A590425); protease/phosphatase inhibitor cocktail (Beyotime Biotechnology, Shanghai, China; Cat# P1045); BCA protein assay kit (Beyotime Biotechnology, Shanghai, China; Cat# P0010S); enhanced chemiluminescence (ECL) substrate (Thermo Fisher Scientific, USA; Cat# NCI5079).

Primary Antibodies: Anti-p65 (Proteintech, Rosemont, IL, USA; Cat# 10745-1-AP; dilution 1:3000); anti-TNF-*α* (Proteintech; Cat# 60291-1-IG; dilution 1:3000); anti-IL-6 (Affinity Biosciences, Cincinnati, OH, USA; Cat# DF6087; dilution 1:1000); anti-*β*-actin (Proteintech; Cat# 20536-1-AP; dilution 1:2000); HRP-conjugated secondary antibodies (dilution 1:1000).

Cells: Human immortalized chondrocytes (SV40-transfected; Meisen CTCC, Zhejiang, China; Cat# CTCC-001-0662) were cultured in complete growth medium (Meisen CTCC; Cat# CTC-001-0662-CM) at 37 °C in a humidified atmosphere with 5% CO₂.

Animals: A total of 18 male specific pathogen-free (SPF) Sprague–Dawley (SD) rats (weighing 180–220 g) were used in this study. All animals were purchased from the Experimental Animal Center of Hunan Academy of Traditional Chinese Medicine (license number: SYXK [Xiang] 2024–0015). Animals were housed under SPF conditions with controlled temperature (22 ± 2 °C), humidity (55 ± 5%), and a 12-h light/dark cycle, with free access to food and water. All animal experiments in this study were performed in strict accordance with the ARRIVE guidelines (Animal Research: Reporting of *In Vivo* Experiments). The study protocol was approved by the Experimental Animal Ethics Committee of Hunan Academy of Traditional Chinese Medicine (approval number: SY2024-0046, China). All efforts were made to minimize animal suffering and the number of animals used.

### 2.2 LC–MS analysis of major components in BGJXF

For LC–MS analysis, BGJXF samples were thoroughly mixed to ensure homogeneity. An aliquot of 200 μL was transferred to a 1.5 mL microcentrifuge tube, and 600 μL of HPLC-grade methanol was added. The mixture was vortexed vigorously to achieve complete extraction of small molecules, then evaporated to dryness under vacuum. The dried residue was reconstituted in 100 μL of 50% methanol (v/v, methanol:water), vortexed to ensure complete dissolution, and filtered through a 0.22 μm organic membrane to remove particulates. Methanol was selected as the extraction solvent for its efficiency in precipitating proteins and extracting both hydrophilic and moderately hydrophobic constituents from the complex BGJXF matrix. Reconstitution in 50% methanol balances compound solubility and injection compatibility for LC–MS analysis. All sample preparation steps were performed at room temperature to minimize analyte degradation and ensure reproducibility.

Chromatographic separation was conducted on an UltiMate 3,000 UHPLC system (Thermo Fisher Scientific) using an ACQUITY UPLC HSS T3 column (1.8 μm, 2.1 × 100 mm, Waters), with the column temperature maintained at 40 °C and an injection volume of 3 μL. The mobile phase for positive ion mode consisted of 0.1% formic acid in water (A) and 0.1% formic acid in acetonitrile (B); for negative ion mode, phase A was 2 mM ammonium acetate in water and phase B was acetonitrile. The gradient program was as follows: 0–1.5 min, 95% A; 1.5–2.5 min, 95–90% A; 2.5–14 min, 90–60% A; 14–25 min, 60–5% A; 25–30 min, 5–95% A, with a 5-min post-run for column re-equilibration.

Mass spectrometric detection was performed on an AB Sciex 5,600 + TripleTOF instrument, using information-dependent acquisition (IDA) controlled by Analyst TF 1.7 software in both positive and negative electrospray ionization (ESI) modes. The most intense ions (intensity >100 cps) were selected for MS/MS in each cycle. MS^1^ scan range was m/z 50–1,200; collision energy was set at 30 eV, with a maximum of 10 candidate ions per cycle and an accumulation time of 50 ms per MS/MS spectrum. ESI source parameters were: ion source gas 1 (GS1), 60 psi; ion source gas 2 (GS2), 60 psi; curtain gas (CUR), 35 psi; temperature (TEM), 650 °C; ion spray voltage (ISVF), +5,000 V (positive) or −4,000 V (negative).

Raw data files were converted to .abf format using AnalysisBaseFileConverter. Peak detection, alignment, and deconvolution were performed in MS-DIAL (version 4.6). Compound identification was achieved by matching acquired MS^1^ and MS/MS spectra to integrated databases (Metlin, MassBank, MoNA, and HMDB v6.0), with mass tolerances set to 0.01 Da for MS^1^ and 0.05 Da for MS^2^, and an identification score cutoff of 60%. Alignment parameters included an MS^1^ mass tolerance of 0.01 Da and retention time tolerance of 0.2 min. For each identified compound, standard information including PeakID, compound name, retention time (RT), precursor m/z, molecular weight, adduct type, SMILES, InChIKey, database IDs, and spectral match scores is provided in the LC/MS identification dataset deposited in Figshare (DOI: 10.6084/m9.figshare.30738803). Herbal attribution for each compound was assigned based on literature and database resources, as indicated in the supplementary tables. This comprehensive workflow ensures reliable and reproducible identification and assignment of chemical constituents in BGJXF.

### 2.3 Network pharmacology analysis

Active ingredient target prediction: The main ingredients identified by LC–MS were entered into the PubChem database^1^ to retrieve their SMILES strings or three-dimensional structures. Afterwards, these structures were submitted to the SwissTargetPrediction database^2^ for target prediction. In SwissTargetPrediction, the species was set to “*Homo sapiens*,” and only predicted targets with a probability score greater than zero were retained. The remaining predicted targets were standardized to official gene symbols using the UniProt database.^3^

Disease target collection: Using “KOA” as the keyword, targets related to KOA were searched in the following databases: GeneCards,^4^ Online Mendelian Inheritance in Man (OMIM),^5^ and DisGeNET.^6^ In GeneCards, only targets with a correlation score ≥ 10 were included. Targets with a correlation score ≥ 10 in GeneCards were selected. Targets in all databases were merged and duplicates were removed.

Potential target identification and “component-target” network: The predicted component targets and collected KOA disease targets were imported into the “Draw Venn Diagram” tool^7^ to identify common targets (intersections) that represent potential therapeutic targets for BGJXF in treating KOA. A Venn diagram was generated. The components corresponding to these common targets were identified, and the “component-target” network was constructed and visualized using Cytoscape software (version 3.8.2).

To ensure biological and clinical relevance, only targets that were both predicted to interact with BGJXF active ingredients and directly implicated in KOA pathogenesis were included in subsequent analyses. This intersection-based strategy yielded 62 common targets, which served as the core set for downstream network and enrichment analyses, thereby improving specificity and translational significance.

Protein–protein interaction (PPI) network construction: The 62 common targets were submitted to the STRING database (https://www.string-db.org; version 11.5) under “Multiple Proteins,” the organism was set to “*Homo sapiens*,” and a minimum required interaction score (“high confidence”) of 0.700 was used. Disconnected nodes were hidden. The network was visualized and further analyzed using Cytoscape (version 3.8.2), and network topology parameters such as degree, betweenness, and closeness centrality were calculated to identify hub proteins potentially critical for BGJXF’s pharmacological activity.

Gene Ontology (GO) and Kyoto Encyclopedia of Genes and Genomes (KEGG) pathway enrichment analysis: The 62 common targets were uploaded to the DAVID database,^8^ with the identifier set as OFFICIAL_GENE_SYMBOL and species as *Homo sapiens*. GO enrichment covered biological process (BP), cellular component (CC), and molecular function (MF) domains, while KEGG pathway analysis revealed significantly enriched signaling pathways (Benjamini-Hochberg-adjusted *p* < 0.05). Enrichment results were visualized using the Bioinformatics online platform,^9^ and top terms and pathways were selected for further interpretation and discussion. This integrative multi-step approach ensures that the network pharmacology findings are robust, biologically meaningful, and clinically relevant to the mechanisms of BGJXF against KOA.

### 2.4 Molecular docking

Molecular docking simulations were performed using the CB-Dock2 server.^10^ CB-Dock2 is an advanced version of CB-Dock that automates the docking process by predicting binding sites, calculating binding site cavities, and performing molecular docking. CB-Dock2 uses the CurPocket cavity detection method based on protein surface curvature to determine the docking region. For detailed methodology, please refer to the relevant literature. Based on the network pharmacology results, the core targets selected for docking were IL-6 (PDB ID: 8YWP), CCND1 (PDB ID: 6PE8), MMP9 (PDB ID: 1ITV), and BCL2 (PDB ID: 6FBX). Before docking, the CB-Dock2 server automatically removes water molecules and other heteroatoms from the downloaded protein structures (.pdb format, from the RCSB Protein Data Bank, https://www.rcsb.org). These proteins act as receptors. The 3D structures of the ligands (dehydrocorydaline and other key BGJXF components) were downloaded in SDF format from PubChem.^11^

### 2.5 Cell experiment: validation of anti-inflammatory effects of dehydrocorydaline in KOA

Cell culture and treatment: An immortalized human chondrocyte cell line (SV40; Zhejiang Meisen, CTCC-001-0662), representing healthy, non-osteoarthritic chondrocytes, was used for all *in vitro* experiments. Cells were cultured in DMEM/F12 complete medium (Zhejiang Meisen, CTCC-001-0662-CM) containing 10% fetal bovine serum (FBS) and 1% penicillin–streptomycin, and maintained at 37 °C in a humidified 5% CO₂ incubator. Cells at passages 2–6 were used in all assays. Cells were divided into three groups (*n* = 3 biological replicates per group): Control group: normal culture; LPS model group: treated with 10 μg/mL LPS for 24 h; Dehydrocorydaline intervention group: treated with 10 μg/mL LPS for 24 h, followed by 50 μM dehydrocorydaline for 48 h.

Cell treatment: Cells were digested with trypsin, and the digestion time was monitored under a microscope until the cells became round and fell off. Then, the cells were seeded in 6-well plates (NEST, Cat. No. 703001) at a density of 1 × 10^5^ cells/well. Treatment was initiated when cells reached approximately 90% confluence. LPS working solution was prepared in complete medium. Dehydrocorydaline was dissolved in DMSO and the final concentration of DMSO in the medium was maintained at ≤0.1% (v/v).

Protein extraction and quantification: Cells were lysed on ice for 30 min using pre-chilled RIPA lysis buffer supplemented with 1 mM PMSF and 1 × protease/phosphatase inhibitor cocktail. Lysates were centrifuged at 12,000 rpm for 15 min at 4 °C (Eppendorf 5415R centrifuge). Supernatants were collected and protein concentrations were determined using a BCA assay kit (Beyotime, Cat. No. P0010S). Protein concentrations were adjusted to the same level for subsequent analysis.

Western Blotting: Gel electrophoresis: Proteins were separated by SDS-PAGE (100 μg per lane). The appropriate separation gel concentration was selected according to the molecular weight of the target protein: 10% for p65 (65 kDa), 15% for TNF-*α* (17 kDa), and 12% for IL-6 (24 kDa). All samples were run on a 5% stacking gel. The electrophoresis voltage was constant: 80 V for stacking gel and 120 V for separation gel (Tanon EPS300).

Membrane transfer: Proteins were transferred to a 0.45 μm PVDF membrane (Millipore) using a wet transfer method with a constant current of 100 mA.

Blocking and antibody incubation: The membrane was blocked with 5% (w/v) skim milk powder (Yili) in TBST for 2 h at room temperature. Primary antibodies were diluted in blocking buffer as follows: anti-NF-κB p65 (1:3,000, Proteintech, 10,745-1-AP), anti-TNF-α (1:3,000, Proteintech, 60,291-1-IG), anti-IL-6 (1:1,000, Affinity, DF6087), and anti-*β*-actin (1:2,000, Proteintech, 20,536-1-AP). Membranes were incubated with primary antibodies overnight at 4 °C. After washing, membranes were incubated with horseradish peroxidase (HRP)-conjugated secondary antibodies (goat anti-rabbit IgG or goat anti-mouse IgG, ZSGB-BIO, China) at a dilution of 1:5,000 at 37 °C for 2 h.

Detection: Protein bands were developed using ECL substrate (Thermo, Cat. No. NCI5079) and imaged using a chemiluminescent detection system.

Statistical analysis: Band intensity was quantified using ImageJ software. The expression levels of target proteins were normalized to β-actin. Statistical significance was assessed using one-way analysis of variance (ANOVA) and Tukey *post hoc* test in SPSS software (version 23.0). *p* values < 0.05 were considered statistically significant.

### 2.6 Animal model: validation of BGJXF therapeutic effect on KOA

#### 2.6.1 Animal model establishment and drug administration

Male SD rats were randomly divided into a normal control group (*n* = 6) and a KOA model group (*n* = 12) after adaptive feeding for 1 week. The KOA model was established in the right knee joint according to the literature method. The model group rats were injected with 0.2 mL papain solution (40 g·L-1, 40 mg/mL) into the right knee joint cavity on the 1st, 4th and 7th days; the normal control group was injected with an equal volume of sterile saline into the joint cavity on the same day. The rats with successful modeling (*n* = 12) were randomly divided into two groups on the 8th day: model group (*n* = 6): daily gavage with an equal volume of saline. BUGUJXUE group (*n* = 6): daily gavage with BUGUJXUE, the dose was 56.4 mg·kg^−1^ body weight. Normal control group (*n* = 6): daily gavage with saline, continuous treatment for 28 days, once a day. The dose (56.4 mg·kg^−1^ per day) and treatment duration (28 days) of BGJXF were established based on clinical equivalent dose conversion using the body surface area normalization method recommended by the Chinese Pharmacopoeia, as well as relevant literature reports regarding the application of traditional Chinese medicine formulas in animal models of KOA.

#### 2.6.2 Sample collection and processing

After 28 days of treatment, the rats were anesthetized and blood was collected from the abdominal aorta. The whole blood was allowed to stand at room temperature for 1 h, centrifuged at 4 °C for 10 min (3,500 rpm), and serum was collected. The serum was stored at −80 °C after aliquoting. The right knee joints were dissected, and 4 knee joints were randomly selected from each group and fixed with 10% neutral formalin buffer for histological analysis. The remaining knee joint tissues were quickly frozen and stored at −80 °C for further analysis.

#### 2.6.3 ELISA detection of serum IL-1β and IL-6 levels

The commercially available ELISA kits were used to determine the serum IL-1β and IL-6 levels according to the kit instructions.

#### 2.6.4 Knee joint tissue morphology observation (H&E staining)

The formalin-fixed knee joint tissues were decalcified in 10% ethylenediaminetetraacetic acid (EDTA, pH 7.4) at room temperature for 21 days with regular solution changes, dehydrated, paraffin-embedded and sectioned. Serial sections were stained with hematoxylin–eosin (H&E) according to standard methods. The stained tissue morphology was observed under an optical microscope to evaluate the pathological changes of articular cartilage, synovium and subchondral bone.

### 2.7 Statistical analysis

All quantitative data are presented as mean ± standard deviation (SD). Statistical analyses were performed using SPSS software (version 23.0, IBM, USA). Comparisons among multiple groups were conducted using one-way analysis of variance (ANOVA) followed by Tukey’s *post hoc* test for multiple comparisons. Differences were considered statistically significant at *p* < 0.05. Graphs were generated using GraphPad Prism 8.0 (GraphPad Software, USA).

## 3 Results

### 3.1 Chemical composition profile of BGJXF

**Figure 1:**
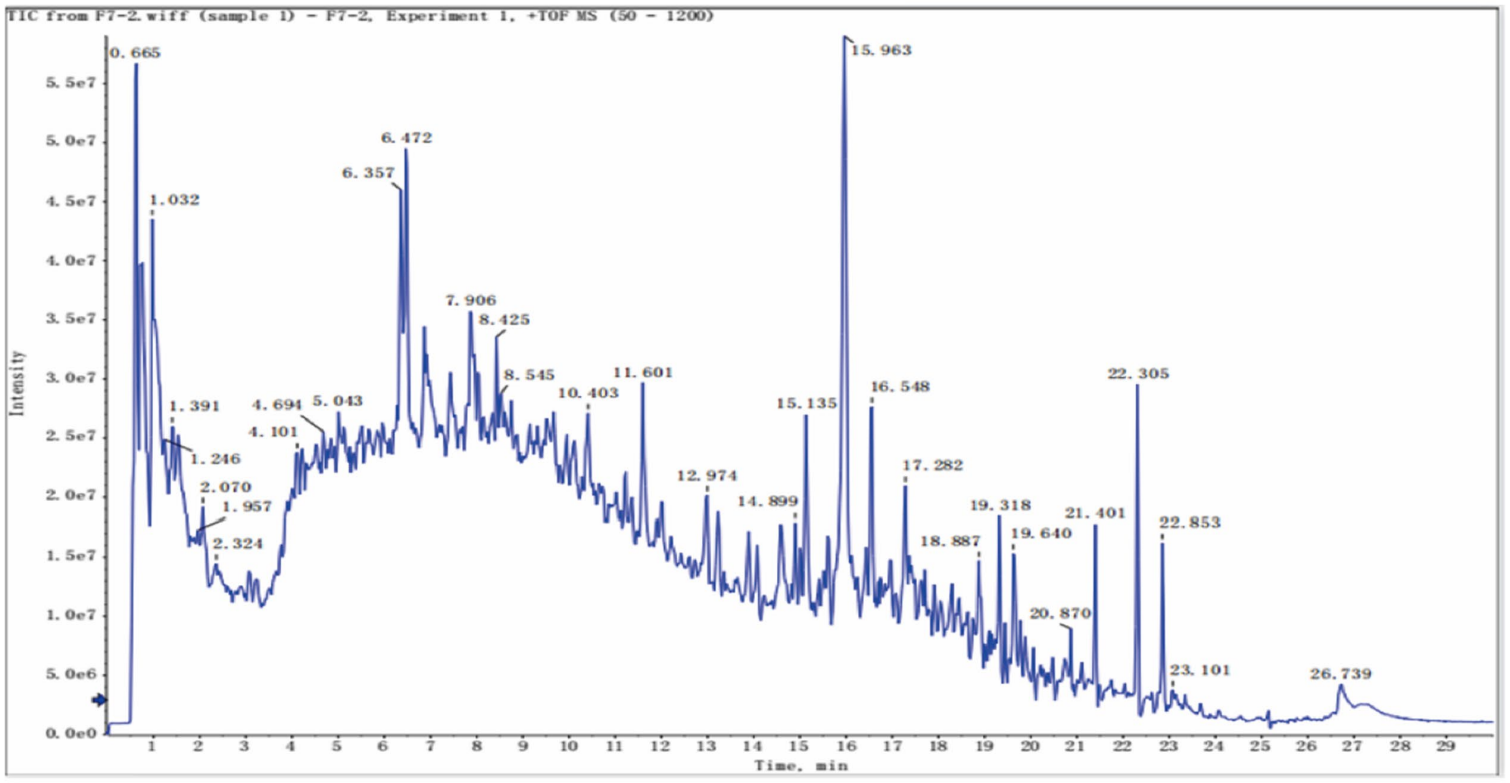
Identification of Main Components (Positive Ions) in Bu Gan Jian Ji Fang.

**Figure 2:**
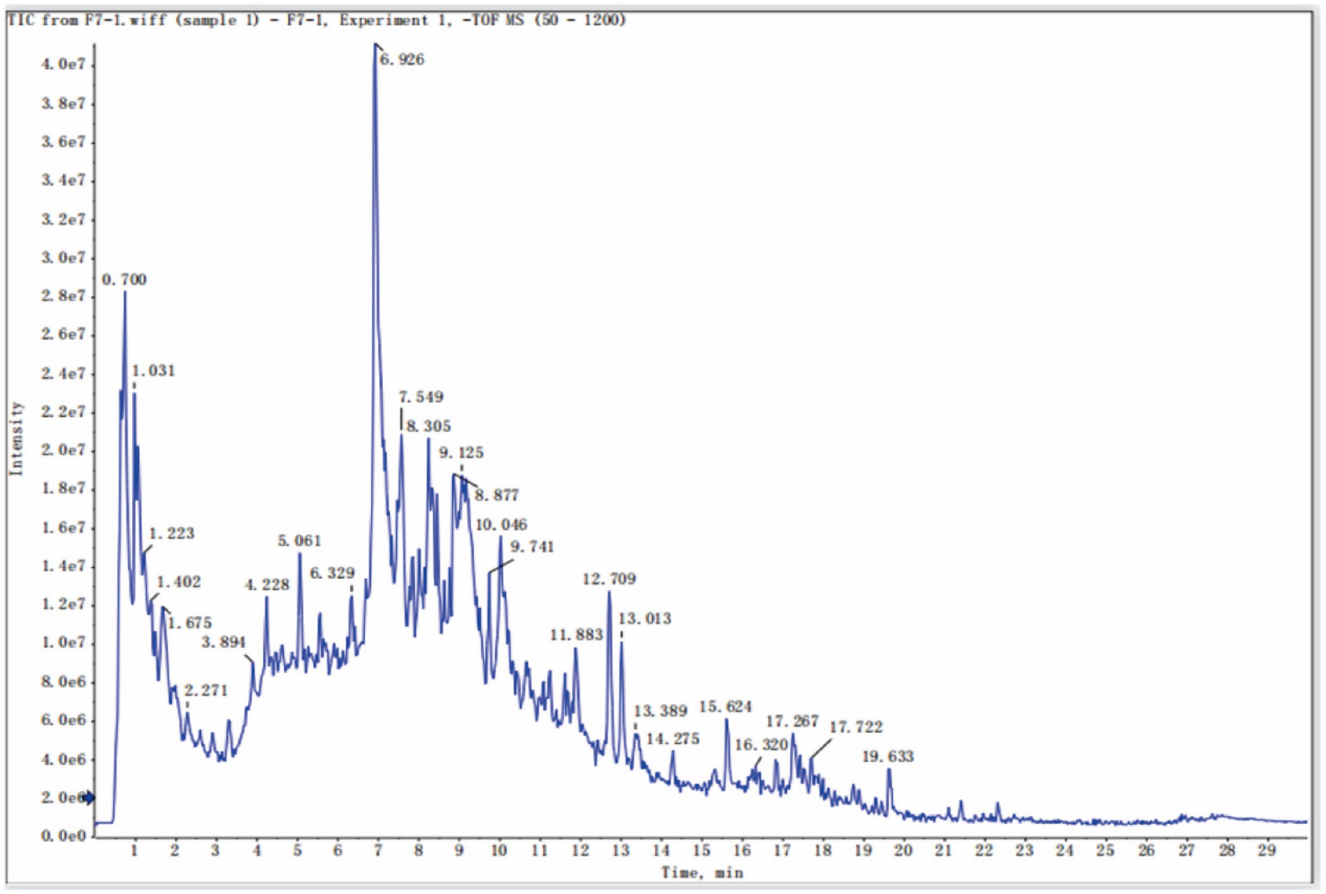
Identification of Main Components (Positive Ions) in Bu Gan Jian Ji Fang.

Liquid chromatography-mass spectrometry (LC–MS) was used to characterize the chemical components of BGJXF. Total ion chromatograms (TIC) were obtained in positive ion mode (+TOF MS) and negative ion mode (-TOF MS). The analysis results showed that a total of 91 compounds were identified in BGJXF (see Supplementary Table S1 for details). All compound identification was performed in the laboratory of Hunan Academy of Traditional Chinese Medicine using a validated LC–MS-based untargeted metabolomic workflow. For sample preparation, the BGJXF extract was homogenized, and 200 μL was combined with 600 μL methanol, evaporated to dryness, reconstituted in 100 μL 50% methanol, and filtered through a 0.22 μm membrane. LC–MS data were acquired in both positive and negative ion modes using an UltiMate 3,000 UHPLC system (Thermo Fisher Scientific) coupled to a 5,600 QTOF high-resolution mass spectrometer (AB SCIEX) with an ACQUITY UPLC HSS T3 column (1.8 μm, 2.1mm × 100 mm, Waters). Data processing was performed using MS-DIAL (v4.6) for peak detection, alignment, and deconvolution, with both MS^1^ and MS/MS spectra matched against integrated databases (Metlin, MassBank, MoNA, HMDB v6.0). Strict mass tolerances (MS^1^: 0.01 Da, MS^2^: 0.05 Da) and a minimum identification score of 60% were applied. For each identified compound, standard information—including PeakID, compound name, retention time (RT), precursor m/z, molecular weight, adduct type, SMILES, InChIKey, database IDs, and spectral match scores—is provided in Supplementary Table S1 (positive mode) and Supplementary Table S2 (negative mode). The herbal attribution of each compound was assigned based on literature and database resources and is indicated in the Supplementary tables. This comprehensive workflow ensures reliable and reproducible identification and assignment of chemical constituents in BGJXF. Total ion chromatograms (TIC) were obtained in positive ion mode (+TOF MS) and negative ion mode (–TOF MS). In positive ion mode TIC (Figure 1), multiple peaks were observed with retention times ranging from 1 to 29 min. The peak intensity of the strongest peak was approximately 6.0 × 10^7^ counts. In negative ion mode TIC (Figure 2), multiple peaks were detected with peak intensities as high as 4.0 × 10^7^ counts. These different TIC spectra indicate that BGJXF contains multiple chemical components and exhibits different chromatographic behaviors in the two ionization modes.

### 3.2 Network pharmacology analysis

**Figure 3:**
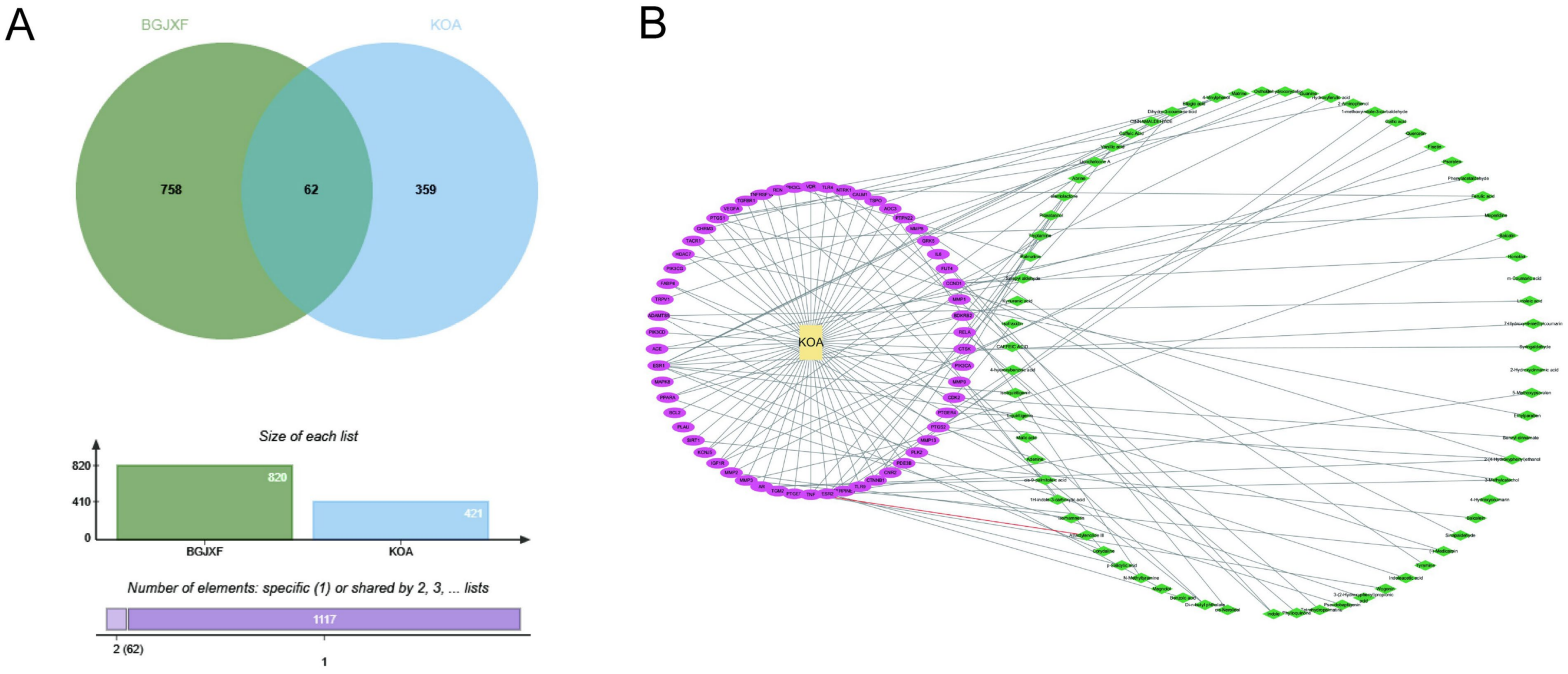
Network pharmacology analysis of Bugan Jianxi Formula **(A)** Venn diagram of targets for BGJXF treatment of KOA **(B)** Visualization of BGJX components and KOA interaction through network interaction graph Functional Enrichment Analysis.

We used network pharmacology to elucidate the relationship between the active ingredients of BGJXF and disease targets, aiming to reveal its potential mechanism for treating KOA. The Venn diagram shows the intersection between BGJXF targets (green, 758 targets) and KOA disease targets (blue, 421 targets), revealing 62 common targets (Figure 3A). This suggests that there is a specific interaction between the active ingredients of BGJXF and the pathogenesis of KOA. The network visualization depicts KOA as the central yellow node, purple nodes represent the active ingredients of BJXF, and green nodes represent their corresponding targets. The connecting lines clearly depict the interactions between KOA, BJXF ingredients, and targets. The network suggests that BJXF may exert its therapeutic effects on KOA by regulating these 62 common targets (Figure 3B).

To further validate the biological and clinical relevance of these findings, we focused on the 62 intersecting targets that are both predicted to be regulated by BGJXF active components and directly implicated in KOA pathogenesis. This intersection-based strategy enhances the specificity and translational value of the analysis and provides a focused core for subsequent network and enrichment studies. The 62 common targets were used to construct the protein–protein interaction (PPI) network and subjected to functional annotation via Gene Ontology (GO) and Kyoto Encyclopedia of Genes and Genomes (KEGG) pathway enrichment analyses. This integrative approach enables a comprehensive exploration of the underlying mechanisms by which BGJXF may exert therapeutic effects on KOA.

**Figure 4:**
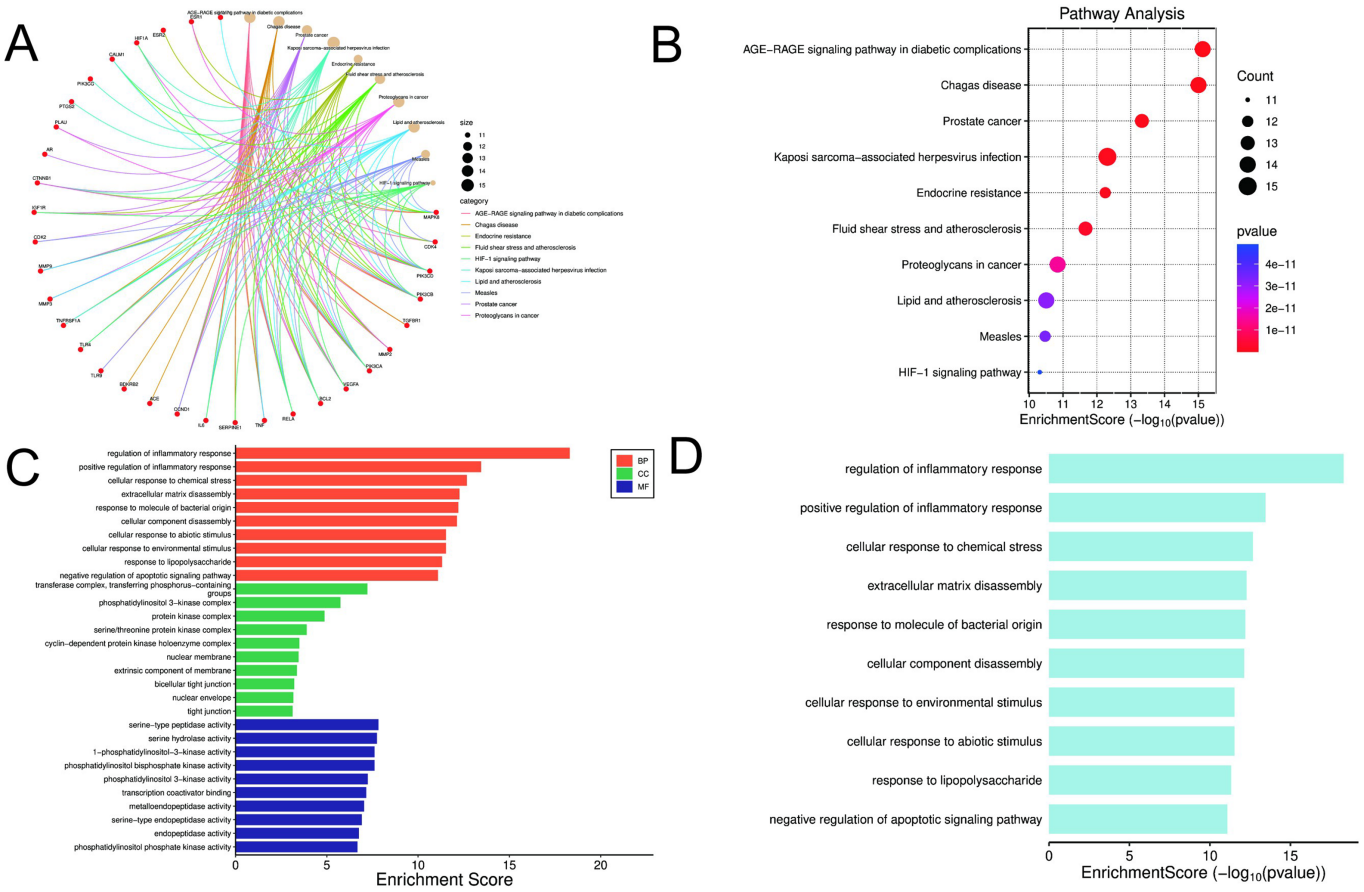
Network and enrichment analyses of BGJXF targets in KOA pathology. **(A)** Target-Pathway Interaction Network, **(B)** KEGG Pathway Enrichment, **(C)** GO Functional Enrichment, **(D)** Top 10 Enriched BPs.

To further elucidate the mechanism of BGJXF in KOA, we constructed a target-pathway interaction diagram to analyze the network interactions between key targets and signaling pathways. The red circular nodes represent targets (e.g., ESR1, HIF1A, PIK3CG, PTGS2, CTNNB1), which vary in size (range: 11–15) and are mainly located on the left and bottom. Lines of various colors (red, orange, yellow, green, blue, purple) radiate from these target nodes to the pathway area on the upper right, including the AGE-RAGE signaling pathway in diabetic complications, prostate cancer, HIF-1 signaling pathway, etc. The legend on the right associates the line color with a specific pathway category and node size. The network clearly shows the direct interactions between BGJXF targets and multiple pathways, suggesting that these core interactions may be involved in the pathology of KOA by regulating related pathways (Figure 4A). KEGG pathway enrichment analysis of potential BGJXF targets associated with KOA showed significantly enriched pathways. The X-axis of the Fig. (white background) represents the enrichment score (−log₁₀[*p*-value]), and the Y-axis represents the pathway/disease name (e.g., AGE-RAGE signaling pathway in diabetic complications, Kaposi’s sarcoma-associated herpes virus infection, endocrine resistance, fluid shear stress and atherosclerosis, proteoglycans in cancer, lipids and atherosclerosis, measles, HIF-1 signaling pathway). The data points are mainly red (with a small amount of purple/blue), and their size indicates the number of enriched targets (counts). The legend on the right indicates that the color of the point corresponds to the significance of the p-value, and the black circle indicates the count range 11–15. The results show that the AGE-RAGE and HIF-1 signaling pathways show the most significant enrichment patterns, indicating that BGJXF may intervene in KOA pathology mainly through these pathways (Figure 4B). GO enrichment analysis of potential targets is shown (BP, CC, MF). The bar graphs (white background) show enrichment scores on the x-axis (range: 0–20) and functional terms (e.g., regulates inflammatory response, positively regulates inflammatory response) on the y-axis. The bar graphs are color-coded: orange (BP), green (CC), and blue (MF). BP terms generally exhibit the highest enrichment scores (peaking around 18), while CC and MF scores are lower. This suggests that BGJXF targets are significantly enriched in BP, especially BP associated with inflammation, which may affect KOA pathology (Figure 4C). GO BP enrichment analysis was performed for the top 10 significantly enriched BPs. The bar graph (white background) lists the relevant processes (e.g., regulation of inflammatory response, positive regulation of inflammatory response, cellular response to chemical stress) on the y-axis and the enrichment scores (−log₁₀[*p*-value]) on the x-axis (range: 0–15). All bars are light blue. “Regulation of inflammatory response” showed the highest enrichment score, which further confirmed the finding that BJXF may affect KOA pathology by significantly regulating inflammation-related BPs (Figure 4D).

**Figure 5:**
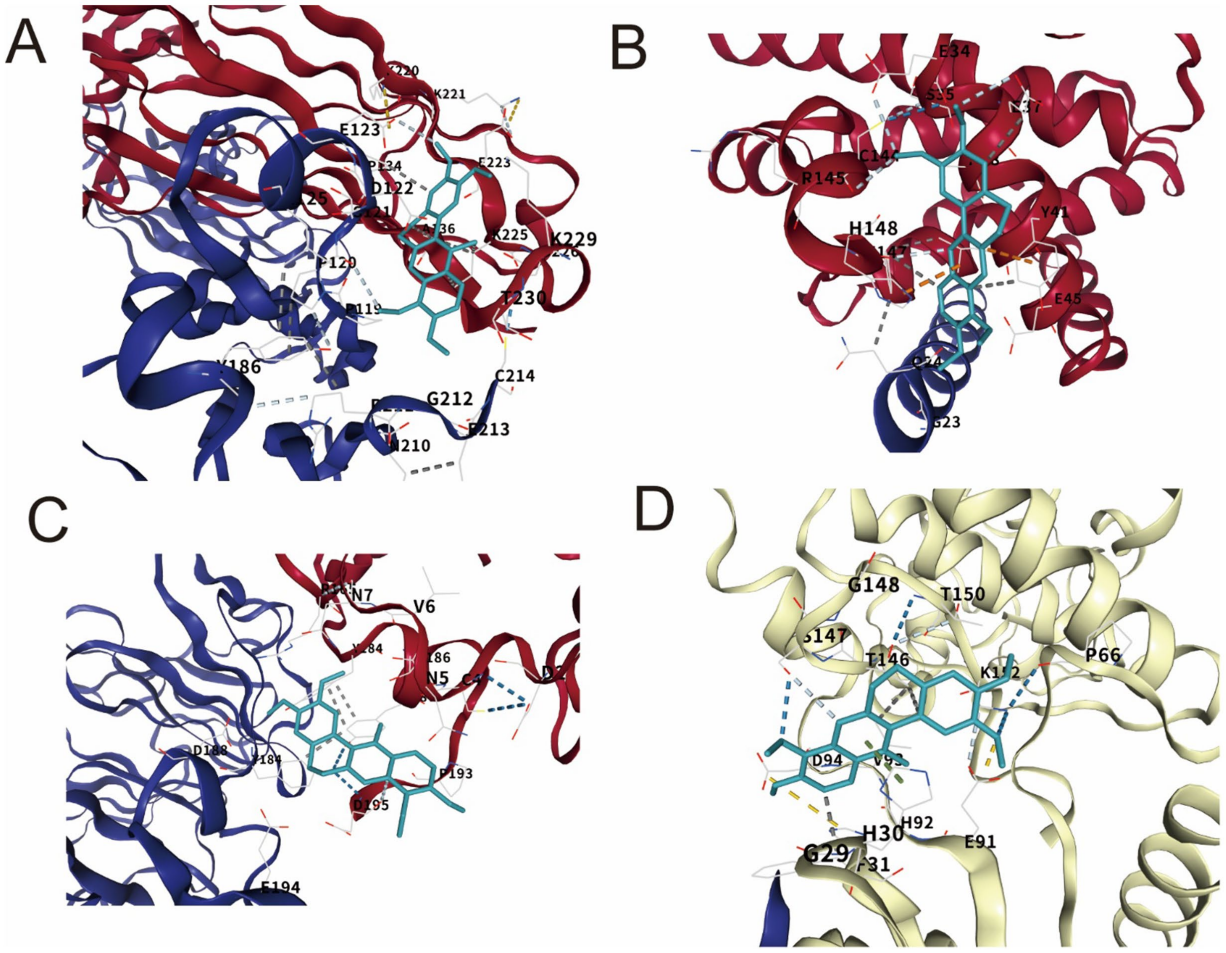
Molecular docking pattern of active components to core targets. **(A)** IL-6- Dehydrocorydaline, **(B)** BCL-2- Dehydrocorydaline, **(C)** MMP9- Dehydrocorydaline, **(D)** CCND1- Dehydrocorydaline.

Based on the results of LC–MS and network pharmacology, dehydrocorydaline was identified as the main active ingredient in BGJX and predicted to be a key component against osteoarthritis (KOA). Therefore, we performed molecular docking simulations to investigate the binding affinity of dehydrocorydaline to core targets (IL-6, BCL2, MMP9, and CCND1). Compared with other representative ingredients of BGJXF, dehydrocorydaline has significantly lower binding energies (indicating higher affinity) for all four targets, as follows: IL-6: −7.4 kcal/mol BCL2: −6.5 kcal/mol MMP9: −7.1 kcal/mol CCND1: −7.2 kcal/mol In comparison, the binding energies of other ingredients range from −6.4 to −7.2 kcal/mol for magnolol, −5.5 to −6.5 kcal/mol for ferulic acid, and −6.4 to −7.1 kcal/mol for palmatine (Table 1). Dehydrocorydaline exhibits the highest affinity for all four targets, indicating that it may be the key active ingredient of BGJXF for the treatment of KOA. To elucidate the spatial binding mode, we visualized the docking posture of dehydrocorydaline within the core target binding pocket: (IL-6-dehydrocorydaline): Dehydrocorydaline (stick model) is embedded within the binding pocket of IL-6 (red/blue ribbon structure) and forms interactions with residues (e.g., E123, F124) (Figure 5A). Dehydrocorydaline makes multi-site interactions with the beige/blue ribbon structure of BCL2 (e.g., residues G29, H30) (Figure 5B). Dehydrocorydaline is precisely located within the red/blue ribbon structure of MMP9 (e.g., residues E91, Z31) (Figure 5C). Dehydrocorydaline binds tightly to the two-color ribbon structure of CCND1 (e.g., residues C145 and D146) (Figure 5D). These visualization results confirmed that dehydrocorydaline stably occupied the binding sites of all four targets, confirming its strong binding affinity predicted by the docking scores.

**Table 1:** Binding energy of the molecular docking between key compounds and core targets (kJ·mol^−1^).

Main components	IL-6	BCL2	MMP9	CCND1
Dehydrocorydaline	−7.4	−6.5	−7.1	−7.2
Magnolol	−6.4	−6.1	−6.6	−6.4
Ferulic acid	−6.2	−5.5	−6.5	−5.6
Palmatine	−7.1	−6.8	−6.4	−7.0

### 3.3 *In vitro* experimental validation

**Figure 6:**
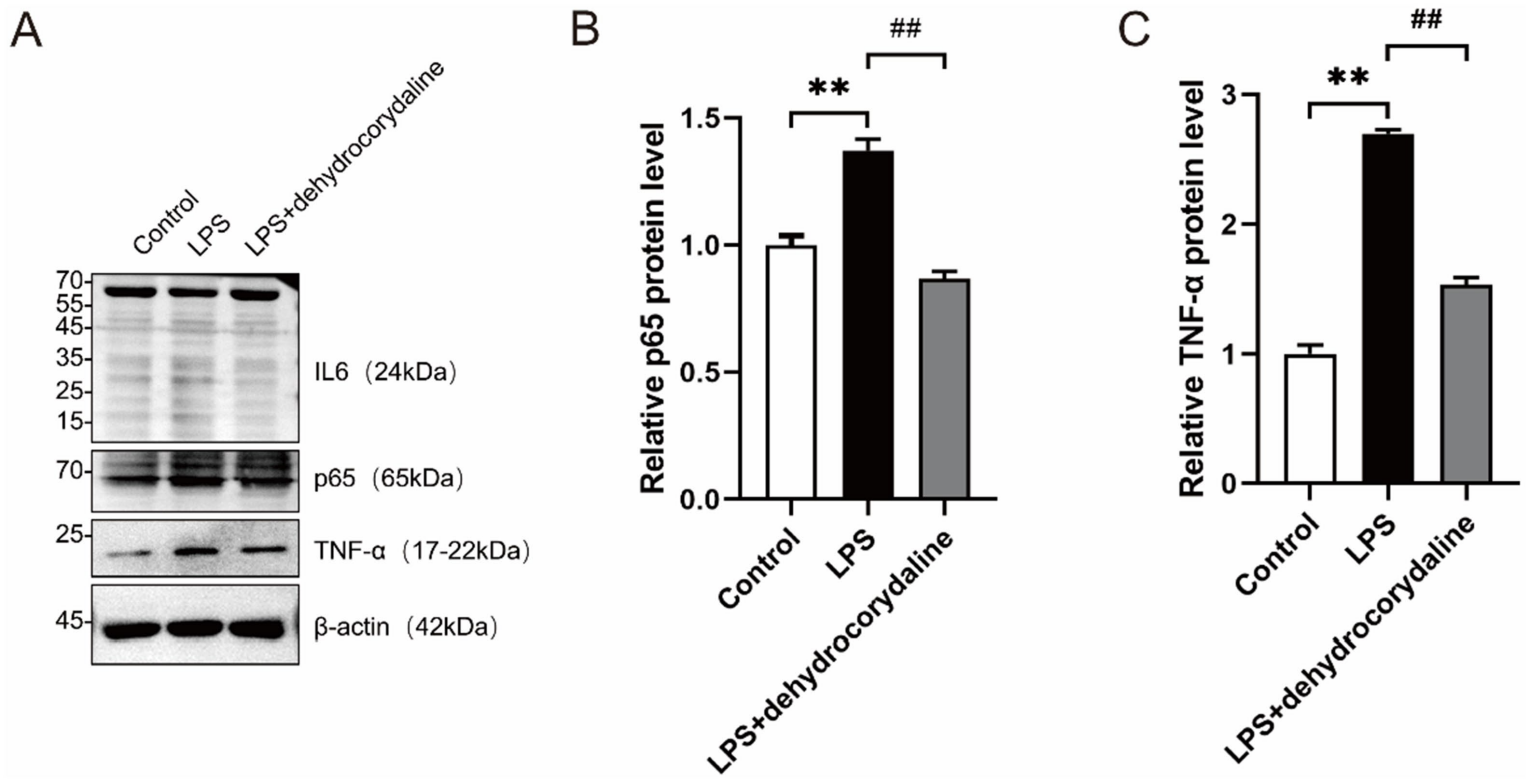
Dehydrocorydaline attenuates LPS-induced upregulation of p65 and TNF-*α* in human chondrocytes. **(A)** Western blot analysis of p65 (~65 kDa), TNF-α (~17 kDa), IL-6 (~24 kDa), and β-actin (loading control, ~42 kDa) in Control, LPS-stimulated (LPS), and LPS + DHC treated chondrocytes. LPS increased p65 and TNF-α levels, rescued by DHC. No specific IL-6 band was detected. **(B)** Quantification of p65 levels (normalized to β-actin). LPS induced a significant increase, significantly attenuated by DHC. **(C)** Quantification of TNF-α levels (normalized to β-actin). LPS induced a significant increase, significantly attenuated by DHC. Data: mean ± SD; *p < 0.05 vs. Control, #p < 0.05 vs. LPS. IL-6 results require validation.

Based on the LC–MS and network pharmacology predictions, the effects of LPS stimulation and dehydrocorydaline intervention on the protein expression of p65, TNF-*α*, and IL-6 in human chondrocytes were evaluated using Western blotting. Protein levels of p65, TNF-α, IL-6, and the loading control *β*-actin were detected across the Control, LPS, and LPS + dehydrocorydaline groups (Figure 6A). Clear bands were observed at the expected molecular weights for p65 (~65 kDa), TNF-α (~17 kDa), and β-actin (~42 kDa). However, a distinct band corresponding to IL-6 (~24 kDa) was not clearly visible, suggesting potential issues related to antibody specificity or low expression levels under the experimental conditions. The band intensities of p65 and TNF-α were markedly increased in the LPS group compared to the Control group, while this increase was attenuated in the LPS + dehydrocorydaline group. Densitometric analysis confirmed a significant increase in relative p65 protein expression in the LPS group compared to the Control group (Figure 6B). Treatment with dehydrocorydaline significantly reduced p65 levels relative to the LPS group. Similarly, densitometric analysis showed a significant increase in relative TNF-α protein expression in the LPS group compared to Control, which was significantly reduced following dehydrocorydaline treatment (Figure 6C). These results indicate that LPS stimulation significantly upregulates p65 and TNF-α expression in human chondrocytes, reflecting an inflammatory response, while dehydrocorydaline effectively inhibits this pro-inflammatory activation. The data for IL-6 require further validation due to the absence of a clear detectable band under the current experimental conditions.

### 3.4 *In vivo* experimental validation

**Figure 7:**
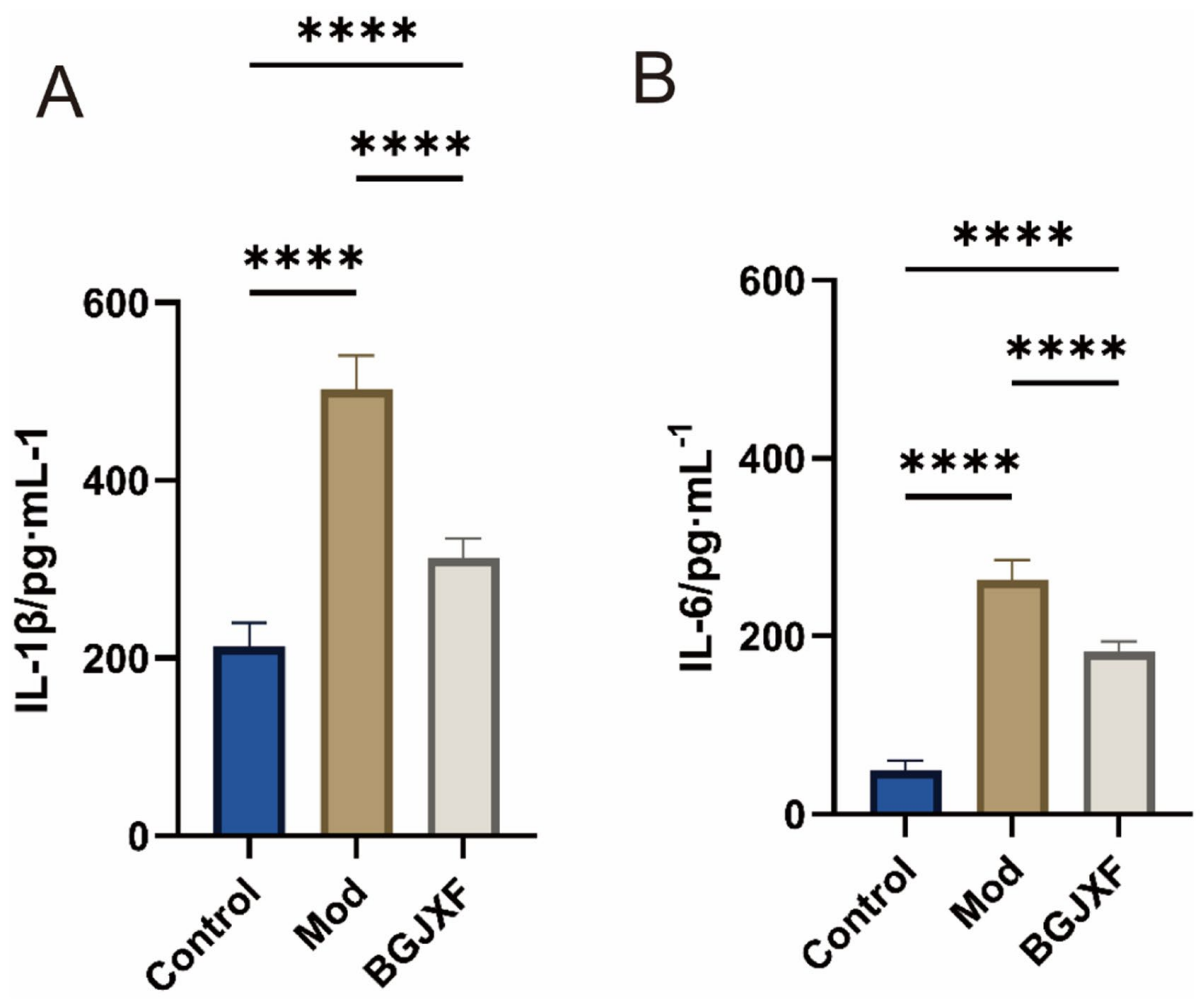
Effects of BGJXF on plasma IL-1β and IL-6 levels in the rat KOA model. **(A)** Plasma IL-1β levels; **(B)** Plasma IL-6 levels. Data are presented as mean ± SD (*n* = 6 per group). Groups: Control (healthy rats), Model (KOA rats + saline), BGJXF (KOA rats + BGJXF, 56.4 mg·kg^−1^·d^−1^ for 28 days). Statistical analysis: One-way ANOVA followed by Tukey’s *post hoc* test. Significance: *p < 0.001 vs. Model group.

The impact of BGJXF on inflammatory cytokine levels was evaluated in the rat KOA model using ELISA to measure plasma concentrations of IL-1β and IL-6 (Figure 7), following the manufacturer’s protocols. Results showed that the levels of both IL-1β and IL-6 were significantly elevated in the Model (Mod) group compared to the Control group. Treatment with BGJXF significantly reduced the levels of these cytokines compared to the Model group (*p* < 0.001, as indicated in the graph).

**Figure 8:**
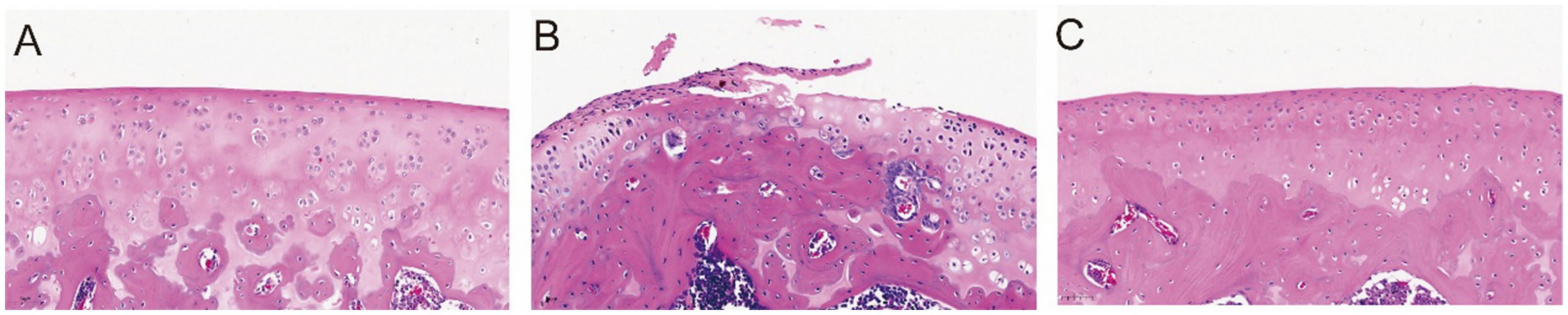
BGJXF ameliorates cartilage degeneration in a rat KOA model as assessed by H&E staining and OARSI scoring: **(A)** Control group: Intact cartilage surface, well-organized chondrocyte distribution, and normal matrix staining (OARSI score = 0). **(B)** Model group: Marked surface erosion, cartilage disorganization, chondrocyte clustering, deep fissures, and inflammatory cell infiltration (OARSI score = 5). **(C)** BGJXF group: Improved cartilage integrity and cellular arrangement, with only mild superficial irregularity and reduced inflammatory infiltration compared to the model group (OARSI score = 2). Scale bar = 20 μm.

These findings suggest that BGJXF may alleviate KOA pathological changes by suppressing inflammatory cytokine production. Data are expressed as mean ± standard deviation (SD) for each group (Control, Model, BGJXF). Differences among groups were analyzed by one-way ANOVA, followed by Tukey’s *post hoc* test for multiple comparisons. Statistical significance was defined as *p* < 0.05. In the bar graph, significance is indicated as follows: “ns” = not significant, *p* < 0.05, *p* < 0.01, *p* < 0.001. All statistical analyses were performed using GraphPad Prism (version 9.5.1). Compared with the Control group, the Model group showed a significant increase in IL-1β and IL-6 levels (*p* < 0.001). Treatment with BGJXF significantly reduced these cytokine levels compared with the Model group (*p* < 0.001), as illustrated in Figure 7. Histopathological assessment of rat knee joint cartilage was performed using hematoxylin and eosin (H&E) staining, and cartilage degeneration was quantitatively evaluated using the OARSI scoring system. Control group: Intact cartilage surface, well-organized chondrocyte distribution, and normal matrix staining were observed (OARSI score = 0) (Figure 8A). Model group: There was marked surface erosion, cartilage disorganization, chondrocyte clustering, deep fissures, and obvious inflammatory cell infiltration, with partial exposure of subchondral bone (OARSI score = 5) (Figure 8B). BGJXF group: Cartilage structure and cellular arrangement were notably improved compared to the model group, with only mild superficial irregularity and reduced inflammatory infiltration (OARSI score = 2) (Figure 8C). These results indicate that BGJXF treatment effectively attenuates cartilage degeneration and histopathological changes in the rat KOA model.

**Figure 9:**
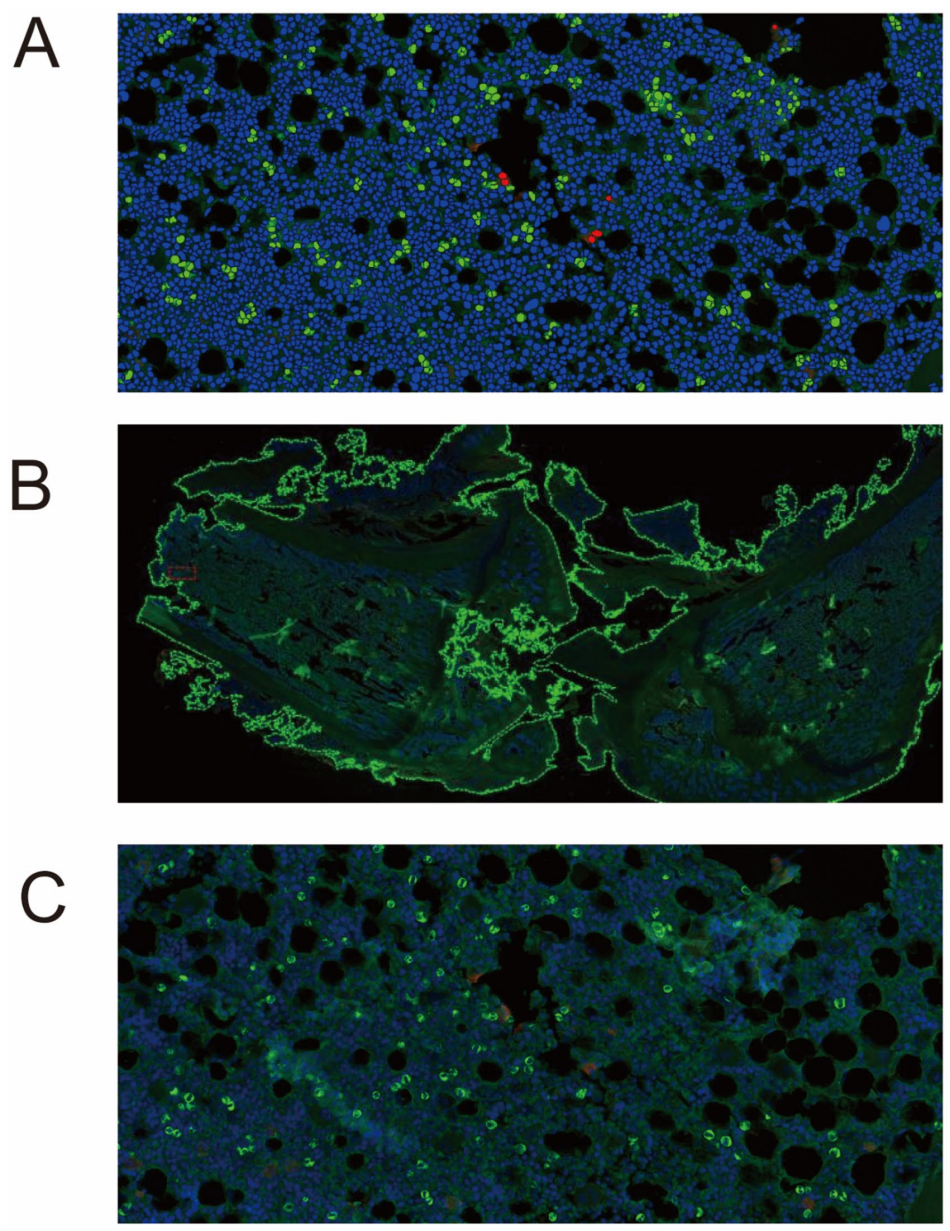
BGJXF reduces IL-6 and p65 expression and co-localization in KOA cartilage. **(A–C)** Representative IF images of rat knee joint cartilage sections from the indicated experimental groups, displaying nuclei (DAPI, blue), IL-6 (green), p65 (green), and merged signals in both whole-tissue and magnified views. Visual analysis indicates a reduction in IL-6 and p65 staining intensity, along with an altered spatial distribution, in the BGJXF group compared to the Model group. Scale bar = 20 μm.

BGJXF Intervention Attenuates IL-6 and p65 Expression and Co-localization in KOA Cartilage. Immunofluorescence (IF) staining was performed to visualize the expression and spatial distribution of IL-6 and p65 within cartilage tissue from the KOA model (Figure 9). Fluorescence micrographs revealed distinct patterns across experimental conditions: Model Group: Exhibited strong and widespread IL-6 and p65 immunofluorescence signals (green) throughout the cartilage tissue. Notably, prominent co-localization (yellow signal in merged channels) of IL-6 and p65 was frequently observed, suggesting a close spatial association between these proteins in the KOA state. BGJXF-treated Group: Demonstrated a marked reduction in both IL-6 and p65 immunofluorescence signal intensity compared to the Model group. The distribution of both proteins appeared less diffuse. Importantly, the co-localization signal (yellow) was substantially diminished in BGJXF-treated samples, indicating a disruption in the spatial association between IL-6 and p65. These representative images provide compelling visual evidence that BGJXF intervention effectively reduces IL-6 and p65 expression within damaged cartilage and disrupts their co-localization pattern observed in the KOA model.

Dehydrocorydaline, a principal alkaloid derived from Corydalis yanhusuo, serves as a representative “minister” component in the BGJXF formula. In the context of traditional Chinese medicine, Corydalis yanhusuo is mainly used to promote Qi and blood circulation and to relieve pain, thereby assisting the “monarch” herbs—*Paeonia lactiflora* (Bai Shao) and *Achyranthes bidentata* (Niu Xi)—which replenish the liver and strengthen tendons and bones. The combination embodies the TCM principle that “when blood moves, wind is extinguished; when Qi moves, dampness is resolved.” Thus, dehydrocorydaline, as a key bioactive compound from the minister herb, works in synergy with the monarch drugs, providing scientific evidence for the rational design and efficacy of multi-component, multi-target TCM formulas.

## 4 Discussion

In this study, we systematically elucidated the multi-target, synergistic mechanisms by which BGJXF alleviates KOA. LC–MS analysis identified 91 bioactive compounds (Figures 1,2), and network pharmacology predicted 62 common targets (Figures 3A,B), suggesting BGJXF may intervene in the dual pathology axis of “glycometabolic stress-hypoxic injury” primarily via the AGE-RAGE and HIF-1 signaling pathways (Figures 4A–D). Molecular docking demonstrated high binding affinity of dehydrocorydaline—a key BGJXF compound—for IL-6, BCL2, MMP9, and CCND1 (Figure 5; Table 1). *In vitro* experiments confirmed that dehydrocorydaline significantly suppressed LPS-induced overexpression of p65 and TNF-*α* in chondrocytes (Figure 6). *In vivo* experiments further validated the efficacy of BGJXF, revealing significant reductions in plasma IL-1β and IL-6 (Figure 7), amelioration of cartilage damage and inflammatory infiltration (Figure 8), and regulation of tissue microenvironment heterogeneity (Figure 9; Table 2). Collectively, these results highlight the core anti-inflammatory and cartilage-protective effects of BGJXF, primarily mediated through inhibition of the NF-κB pathway, and underscore its multi-target, multi-pathway synergistic advantages over single-target agents.

**Table 2:** Quantitative analysis of IL-6 and NF-κB p65 immunofluorescence in rat cartilage from different experimental groups.

Group	IL-6 positive rate (%)	p65 positive rate (%)	IL-6 and p65 double-positive rate (%)	Mean fluorescence intensity of IL-6	Mean fluorescence intensity of p65	IL-6-positive cell density (cells/mm^2^)	p65-positive cell density (cells/mm^2^)
2630CON4-IL-6(570) + P65(520)	0.89	7.39	0.39	80.94	13.20	63	522
2630CON6-IL-6(570) + P65(520)	5.21	3.54	1.17	85.25	12.81	483	328
2630GY4-IL-6(570) + P65(520)	3.60	4.75	1.30	85.64	12.79	275	363
2630GY6-IL-6(570) + P65(520)	6.52	3.08	0.99	89.54	13.64	621	294
2630MOD2-IL-6(570) + P65(520)	0.85	1.88	0.26	78.86	13.52	90	201
2630MOD3-IL-6(570) + P65(520)	0.47	2.22	0.15	78.41	14.00	44	211

KOA, the most prevalent chronic degenerative joint disease worldwide, is characterized by a core pathological feature: a cascade of damage initiated by the imbalance of cartilage homeostasis (25, 26). Recent in-depth investigations into disease mechanisms have broadened the understanding of cartilage injury from a singular “mechanical abrasion” model to a multidimensional pathological process involving metabolic stress, inflammatory storms, and microenvironmental heterogeneity. As the “buffer barrier” of the joint, cartilage integrity depends on the precise regulation of matrix synthesis and degradation by chondrocytes. When chondrocytes undergo phenotypic shifts due to mechanical stress, metabolic dysregulation, or inflammatory stimuli, they initiate a vicious cycle in KOA. Initially, damaged chondrocytes excessively secrete matrix-degrading enzymes, such as MMP-13 and MMP-9, leading to the loss of type II collagen and proteoglycans and resulting in fissures on the cartilage surface. Subsequently, the exposed subchondral bone releases calcium phosphate particles and growth factors (e.g., TGF-β, BMPs), which activate synovial fibroblasts and immune cells, triggering an outburst of pro-inflammatory cytokines such as IL-1β and TNF-*α*. This inflammatory response further exacerbates chondrocyte apoptosis and matrix degradation. Ultimately, subchondral bone remodeling and synovitis work in tandem to drive KOA progression toward its end stage.

Critically, research has demonstrated that the NF-κB signaling pathway plays a pivotal role in the pathogenesis of KOA (27, 28). Activation of NF-κB leads to the release of multiple inflammatory mediators, including TNF-*α*, IL-1β, and IL-6, which not only promote inflammation but also accelerate the degradation of the cartilage matrix (29). Within the intra-articular environment of KOA patients, NF-κB activation is frequently associated with increased chondrocyte apoptosis and elevated expression of matrix metalloproteinases (MMPs), directly contributing to cartilage matrix breakdown (30, 31). Studies using KOA models have shown that NF-κB activation is significantly correlated with increased expression of MMP-13, a key enzyme involved in cartilage degradation (30). Furthermore, NF-κB participates in inflammatory signal transduction by upregulating the expression of inflammation-related genes, thereby exacerbating cartilage damage and inflammatory responses (32). Consequently, therapeutic strategies targeting the NF-κB pathway to suppress synovitis and protect articular cartilage are of paramount importance in the management of KOA.

In recent years, TCM has demonstrated promising efficacy in ameliorating synovitis and cartilage degeneration associated with KOA (24, 25). TCM interventions exert their effects by inhibiting the NF-κB pathway, modulating the MAPK signaling pathway, and influencing cytokine networks, thereby mitigating inflammation and promoting cartilage repair (33, 34). This multifaceted mechanism enables TCM to address multiple aspects of disease progression rather than targeting a single pathway. Additionally, network pharmacology studies have elucidated the synergistic effects of TCM formulas, revealing that interactions among herbal components enhance overall therapeutic outcomes (35). This dual mechanism, suppressing inflammation while promoting cartilage protection, offers novel perspectives for the prevention and treatment of KOA.

Notably, compared with the single-target (IL-1β) inhibition of diacerein, BGJXF exhibits a multi-target synergistic advantage, simultaneously targeting the NF-κB pathway (p65 and TNF-α) and apoptosis-related targets (e.g., BCL2). Its ability to regulate microenvironmental heterogeneity (Figure 9) may provide a novel strategy for addressing the pathological heterogeneity of KOA.

Strengths of this study include the integration of multi-omics approaches—combining comprehensive chemical profiling, network pharmacology, molecular docking, and *in vivo* experimental validation—which together provide a systematic and robust investigation of the therapeutic mechanisms of BGJXF in KOA. The use of both LC–MS–based compound identification and experimental confirmation of key signaling pathways strengthens the reliability and translational potential of our findings. Additionally, the combination of network-level predictions with direct biological assays helps bridge the gap between computational analysis and clinical relevance.

However, this study has several limitations. First, the paradoxical regulation of IL-6, undetectable *in vitro* yet significantly reduced in vivo, suggests that BGJXF may indirectly modulate IL-6 release through the microenvironment, necessitating further investigation into its spatiotemporal kinetics. Second, the functional regulation of targets such as IL-6 and BCL2 by dehydrocorydaline, as well as the dominance of this compound within the formula, remains empirically unverified; targeted monomer interventions or component knockout experiments are required to clarify these roles. Third, the predicted interactions between the AGE-RAGE/HIF-1 pathways and the NF-κB pathway, identified through network pharmacology, lack direct experimental validation. Future research should focus on: (1) utilizing organoid models that integrate the “glycometabolism-hypoxia-inflammation” microenvironment to bridge in vitro-in vivo discrepancies; (2) employing pathway inhibitors or gene-editing techniques to validate the causal contributions of the predicted pathways; and (3) elucidating the dose-dependent and spatiotemporal effects of dehydrocorydaline, along with its synergistic interactions with other components of the formula. In addition, there are several methodological limitations: (1) Absence of a positive control group: Due to experimental resource constraints, a standard pharmaceutical (positive) control group was not included. This omission limits the benchmarking of BGJXF’s efficacy relative to established treatments. Future studies should incorporate a positive control group to provide a more rigorous reference for efficacy assessment. (2) Quantification of cytokines: Although Western blotting was used to confirm protein expression changes *in vitro*, this method may not provide sufficient sensitivity for low-abundance cytokines such as TNF-*α* and IL-6. More sensitive assays, such as ELISA or multiplex immunoassays, are recommended for future studies, particularly in in vitro settings. (3) Histological analysis: The present study used HE staining to assess general tissue morphology; however, specific stains such as Alcian blue, which detects glycosaminoglycans in the cartilage matrix, would have provided more detailed insights into cartilage integrity. Incorporation of such staining methods will be a focus in future work. The theoretical significance of this study lies in providing empirical evidence supporting BGJXF’s “multi-component, multi-target, multi-pathway” synergistic therapeutic approach for KOA, deepening the understanding of the core role of the NF-κB pathway, and emphasizing the importance of microenvironmental modulation and potential synergy with predicted pathways (AGE-RAGE/HIF-1). From a practical perspective, BGJXF and its core component, dehydrocorydaline, demonstrate significant anti-inflammatory and chondroprotective potential, laying a scientific foundation for the development of novel therapeutic strategies targeting inflammatory cascades and stress-induced damage underlying KOA.

## 5 Conclusion

This study systematically elucidated the mechanisms by which BGJXF alleviates KOA through multi-target synergistic actions, integrating data from LC–MS analysis, network pharmacology predictions, and validation through in vitro and *in vivo* experiments. Through the identification of 91 bioactive compounds and the screening of 62 common targets, BGJXF was predicted to intervene in the dual pathology axis of “glycometabolic stress-hypoxic injury,” primarily via the AGE-RAGE and HIF-1 signaling pathways. Molecular docking and in vitro experiments confirmed that dehydrocorydaline, a key potential anti-inflammatory component of BGJXF, exerts its effects by binding with high affinity to crucial targets and significantly inhibiting the p65/TNF-*α* inflammatory cascade within the NF-κB pathway. *In vivo* experiments further validated the overall therapeutic efficacy of the BGJXF formula, demonstrating its ability to significantly reduce plasma levels of IL-1β and IL-6, ameliorate structural cartilage destruction and inflammatory infiltration, and notably regulate tissue microenvironmental heterogeneity.

**Figure 10:**
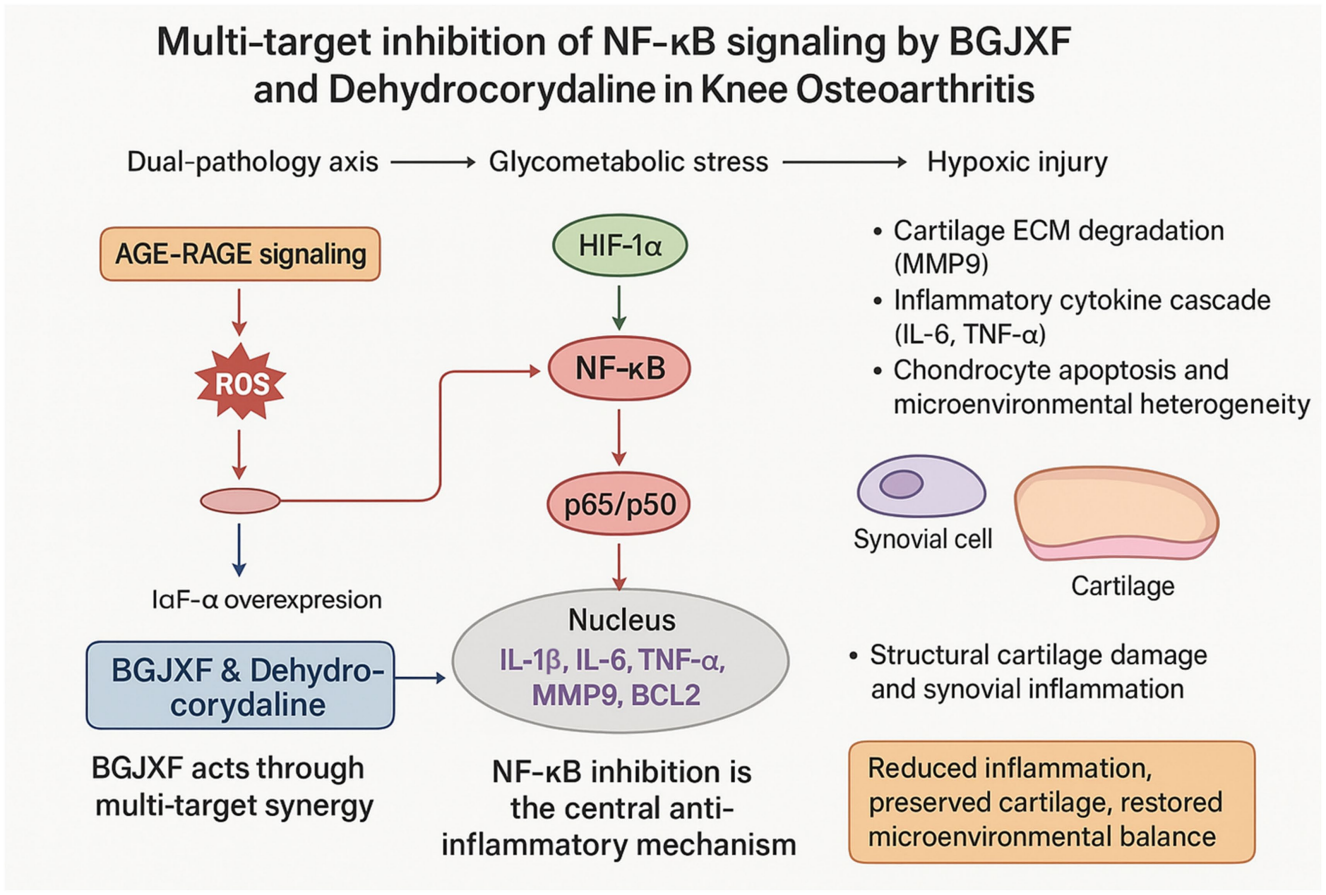
Schematic illustration of the multi-target mechanisms by which BGJXF and dehydrocorydaline alleviate knee osteoarthritis (KOA) via inhibition of NF-κB signaling. BGJXF (Bu Gan Jian Xi Fang) and its key alkaloid component dehydrocorydaline act through a dual-pathology axis involving glycometabolic stress (AGE-RAGE signaling) and hypoxic injury (HIF-1α signaling), both of which converge on the activation of the NF-κB pathway. Inhibition of NF-κB, particularly the p65/p50 subunits, leads to downregulation of pro-inflammatory mediators (IL-1β, IL-6, TNF-α, MMP9, and BCL2), resulting in reduced extracellular matrix (ECM) degradation, attenuation of inflammatory cytokine cascades, decreased chondrocyte apoptosis, and restoration of microenvironmental balance in cartilage and synovial tissues. These coordinated effects culminate in preserved cartilage structure and improved joint function in KOA.

A schematic summary of these multi-target, multi-pathway mechanisms is illustrated in Figure 10. As shown, BGJXF and dehydrocorydaline inhibit NF-κB activation via modulation of the AGE-RAGE and HIF-1α signaling axes, thereby suppressing the expression of key inflammatory mediators (IL-1β, IL-6, TNF-α, MMP9, BCL2), reducing cartilage ECM degradation, inflammation, and chondrocyte apoptosis, and ultimately preserving cartilage structure and restoring microenvironmental balance in knee osteoarthritis.

Importantly, these findings are highly consistent with the TCM theory of “liver deficiency and collateral impediment.” According to this theory, liver blood deficiency leads to insufficient nourishment of the sinews and collaterals, increasing vulnerability to joint degeneration, while collateral obstruction caused by blood stasis and phlegm further aggravates pain and dysfunction. Our experimental results, showing that BGJXF improved cartilage integrity, reduced inflammatory infiltration, and suppressed key pro-inflammatory cytokines, can be regarded as the modern molecular and pathological correlates of ‘nourishing liver blood’ and ‘unblocking the collaterals’ as described in TCM theory. Thus, our data provide mechanistic evidence bridging classical TCM theory and contemporary osteoarthritis pharmacology.

## Data Availability

The original contributions presented in the study are publicly available. The LC/MS identification results generated in this study have been deposited in the Figshare repository. This data can be found at: 10.6084/m9.figshare.30738803.

## Footnotes

1
https://pubchem.ncbi.nlm.nih.gov

2
http://www.swisstargetprediction.ch

3
https://www.uniprot.org

4
https://www.genecards.org

5
https://www.omim.org

6
https://www.disgenet.org

7
https://bioinformatics.psb.ugent.be/webtools/Venn

8
https://david.ncifcrf.gov

9
http://www.bioinformatics.com.cn

10
https://cadd.labshare.cn/cb-dock2/

11
https://pubchem.ncbi.nlm.nih.gov
